# Proteomic profiling of salivary gland after nonviral gene transfer mediated by conventional plasmids and minicircles

**DOI:** 10.1038/mtm.2014.7

**Published:** 2014-04-02

**Authors:** Ramaz Geguchadze, Zhimin Wang, Lee Zourelias, Paola Perez-Riveros, Paul C Edwards, Laurie Machen, Michael J Passineau

**Affiliations:** 1 Gene Therapy Program, Allegheny Health Network, Pittsburgh, Pennsylvania, USA; 2 National Institute of Dental and Craniofacial Research, Bethesda, Maryland, USA; 3 Department of Oral Pathology, Medicine and Radiology, Indiana University School of Dentistry, Indianapolis, Indiana, USA

## Abstract

In this study, we compared gene transfer efficiency and host response to ultrasound-assisted, nonviral gene transfer with a conventional plasmid and a minicircle vector in the submandibular salivary glands of mice. Initially, we looked at gene transfer efficiency with equimolar amounts of the plasmid and minicircle vectors, corroborating an earlier report showing that minicircle is more efficient in the context of a physical method of gene transfer. We then sought to characterize the physiological response of the salivary gland to exogenous gene transfer using global proteomic profiling. Somewhat surprisingly, we found that sonoporation alone, without a gene transfer vector present, had virtually no effect on the salivary gland proteome. However, when a plasmid vector was used, we observed profound perturbations of the salivary gland proteome that compared in magnitude to that seen in a previous report after high doses of adeno-associated virus. Finally, we found that gene transfer with a minicircle induces only minor proteomic alterations that were similar to sonoporation alone. Using mass spectrometry, we assigned protein IDs to 218 gel spots that differed between plasmid and minicircle. Bioinformatic analysis of these proteins demonstrated convergence on 68 known protein interaction pathways, most notably those associated with innate immunity, cellular stress, and morphogenesis.

## Introduction

Gene transfer for therapeutic purposes is now an established and promising treatment strategy in disease paradigms where conventional treatments are unavailable or inadequate. Despite decades of frustratingly slow progress, several recent successes^1–3^ demonstrate the vital and transformative role that gene therapy will play in the future of medicine. In general, the field has progressed beyond proof-of-principle into a new focus on research questions related to clinical practicality. Delivery of genetic payloads remains, as it has always been, the greatest challenge to realizing the full clinical potential of gene therapy. With clinically efficacious gene transfer now a demonstrated reality, current research is exploring more nuanced delivery issues, such as changes in intracellular programming that may occur as the result of gene transfer to target tissues.

Nonviral gene transfer overcomes one of the most vexing challenges to clinical implementation of gene therapy, namely the introduction of viral vector antigens into host cells and tissues.^4–6^ However, due to the vanguard role viral vectors have historically played in advancing gene therapy from the theoretical to clinical reality, research examining host response to nonviral vectors has understandably lagged. Because nonviral vectors lack the protein antigens necessary to initiate classical humoral or cell-mediated extracellular immunity, these vectors have often been assumed to be modestly or negligibly immunogenic provided they encode a therapeutic protein that is native to the host.^7^ This assumption is reasonable as far as it goes, but intracellular host response to nonviral vectors has not been well studied and may present an additional challenge for gene therapy.

Our group has successfully applied the principle of ultrasound-assisted gene transfer (UAGT) to the salivary gland,^8^ relying upon the biophysical effect referred to as sonoporation^9^ to allow a plasmid to physically transit the membranes of salivary gland epithelial cells. This gene transfer model relies upon bloodless cannulation of the salivary duct, and infusion of the vector and microbubbles into the intraductal labyrinth of the salivary gland.^10^ As such, the volume, concentration, and composition of the gene transfer solution can be precisely controlled and isolated from blood or mucosal defenses, allowing us to parse out vector-specific physiological responses in the target tissue. UAGT does not stimulate inflammation or cellular infiltration of the gland, and thus, we can profile the proteome of the homogenized organ without concern for contributions from exogenous cells.

In this study, we took advantage of these characteristics of salivary gland gene transfer as a model system to explore the impact of nonviral gene transfer upon the proteome of the gland. Gel-based proteomic profiling has limitations,^11^ but it provides a global, unbiased look at gene transfer–associated changes in organ physiology as manifested in the proteome. Our goal was to understand the overall magnitude of changes in the proteomic profile of the gland, if any, following UAGT with first-generation plasmids and advanced minicircle^12,13^ vectors. We theorize that subcellular proteomic alterations associated with nonviral gene transfer to the salivary gland will give us broadly generalizable insights into intracellular response to nonviral vectors in a variety of target tissues and thus advance our understanding of the potential for vector-associated intracellular toxicity in gene therapy.

## Results

### Generation of minicircle vectors based upon pCMV-GL3enh

**Figure 1:**
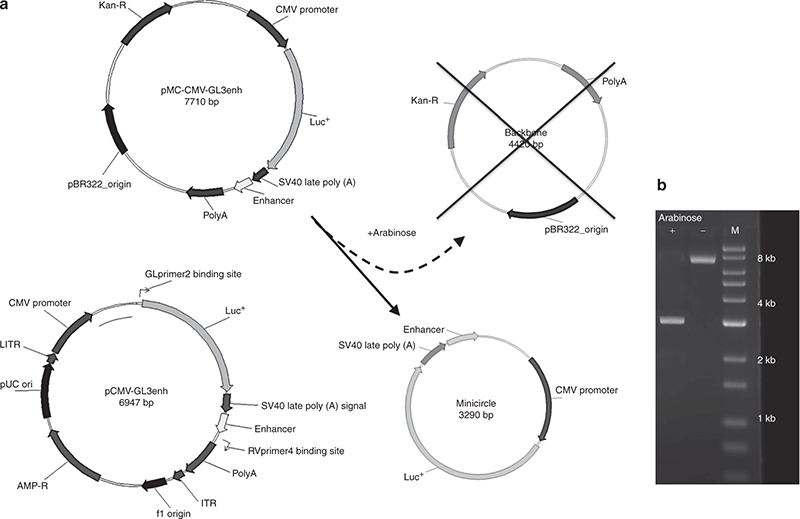
Generation of Minicircle plasmid DNA. (**a**) The expression cassette was excised from the first-generation plasmid vector, pCMV-GL3enh and ligated into the parental plasmid, pMC-Gl3-Enhancer. After the addition of arabinose, the parental vector is cleaved, and the progeny minicircle (C) and backbone, containing the bacterial origin of replication and antibiotic resistance, are religated. The backbone sequence contains several engineered I-SecI restriction sites that ultimately lead to the degradation of the parental DNA but not the Minicircle DNA. (**b**) DNA gel of the minicircle prep shows the intact parental vector (7.7 kb) in the absence (−) of arabinose, and the minicircle in the presence (+) of arabinose (3.29 kb, note the absence of the degraded backbone). Lane M indicates the reference ladder. Plasmids were cut by EcoRV to achieve linearization prior to electrophoresis.

The expression cassette from pCMV-GL3enh was successfully transferred to the pMC.BESPX-MCS1 parental vector and confirmed by sequence analysis. This vector was used to transform the ZYCY10P3S2T bacterial strain, and transformed bacteria were then exposed to arabinose, resulting in excision and recircularization of the cytomegalovirus (CMV)-GL3enh minicircle and the release and degradation of the parental backbone. The structure of the isogenic pCMV-GL3enh plasmid and CMV-GL3enh minicircle are shown in Figure 1a. Figure 1b shows bands of the appropriate size for the CsCl-purified products of the excision reaction, including the 7710bp parental vector and the 3290bp minicircle.

### UAGT to the salivary gland results in global but heterogeneous gene transfer and does not result in cellular infiltration or tissue disruption

**Figure 2:**
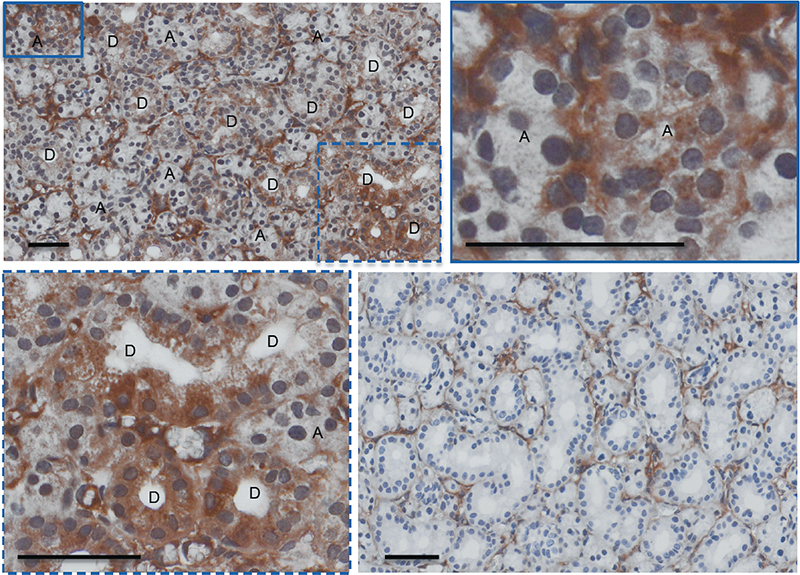
Immunohistochemical analysis of mouse salivary glands 24 hours following ultrasound-assisted gene transfer (UAGT) of a α-1-antitrypsin-expressing plasmid. Sections stained in the presence of the polyclonal anti-A1AT antibody (upper left) show a global but heterogeneous staining pattern that labels both ductal (D) and acinar (A) cells. Sections stained in the absence of the antibody (lower right panel) reveal some background staining in the interstitial connective tissues, but cell bodies are clear of staining. Bar = 50 µm.

In our initial report describing UAGT to the mouse salivary gland,^8^ we reported that the process appeared to be stochastic and without preference to cell type, but our analysis was somewhat confounded by autofluorescence of the salivary gland tissue. To improve our approach, we relied upon HRP-based staining of tissue sections and utilized gene transfer of human α-1-antitrypsin (A1AT) as the marker and the results are shown in Figure 2. Some background staining was observed in the extracellular matrix, but there was no background staining in any of the cellular elements when the A1AT antibody was excluded (right lower panel). Specific staining for the marker transgene was observed in both ductal (left lower panel) and acinar cells (right upper panel). Globally, staining was observed throughout the gland with occasional regions of intense staining (upper left panel).

Additionally, we utilized these sections to evaluate whether UAGT of a foreign gene results in cellular infiltration or tissue disruption of the mouse salivary gland. This question was particularly pertinent to our present study because if cellular infiltration is absent, we can assume that proteomic changes that occur in the tissue as a result of plasmid or minicircle gene transfer are primarily attributable to the intracellular response of native salivary gland cells to the vectors. The lower right panel in Figure 2 shows negative control (*i.e.*, staining without antibody) tissue sections serial to those stained for A1AT, demonstrating a lack of cellular infiltration and no noticeable disruption of tissue architecture following UAGT with a plasmid vector encoding A1AT. Formal scoring of the study tissue for these indices was performed by a blinded oral pathologist (Dr. Paul Edwards) and results indicated no difference between naive control glands and experimental (data not shown).

### Minicircle vectors mediate superior UAGT relative to first-generation plasmids

**Figure 3:**
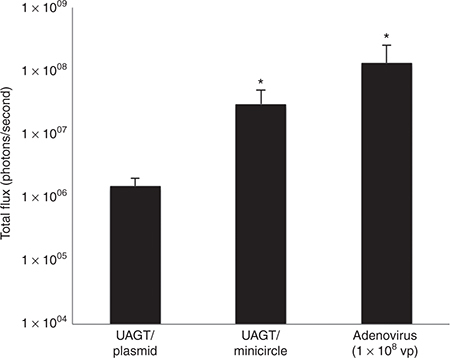
Quantification of luciferase activity 24 hours following gene transfer to the salivary gland. Average total flux (photons/second) is measured by the charge-coupled devices camera system over a 60-second sampling period. Gene transfer with ultrasound-assisted gene transfer (UAGT)/plasmid (*n* = 6), UAGT/minicircle (*n* = 8), and adenovirus at a dose of 1 × 10^8^ viral particles (*n* = 6) is compared. “*” indicates statistically significant differences (*P* < 0.05, Mann–Whitney *U*-test).

In initial testing of our minicircle vectors, we used bioluminescent imaging following UAGT to compare the gene transfer efficiency of the minicircle to our conventional pCMV-GL3enh vector, a vector system that is routinely utilized in our laboratory. Using our standard UAGT conditions, with eqimolar concentrations of pCMV-GL3enh or pMC-CMV-GL3enh, we found that the minicircle was more efficient in the magnitude of transgene expression (see Figure 3). This finding is consistent with an earlier report of a similar phenomenon following electrotransfer to muscle.^14^

### Sonoporation without gene transfer exerts negligible effects upon salivary gland protein expression

**Figure 4:**
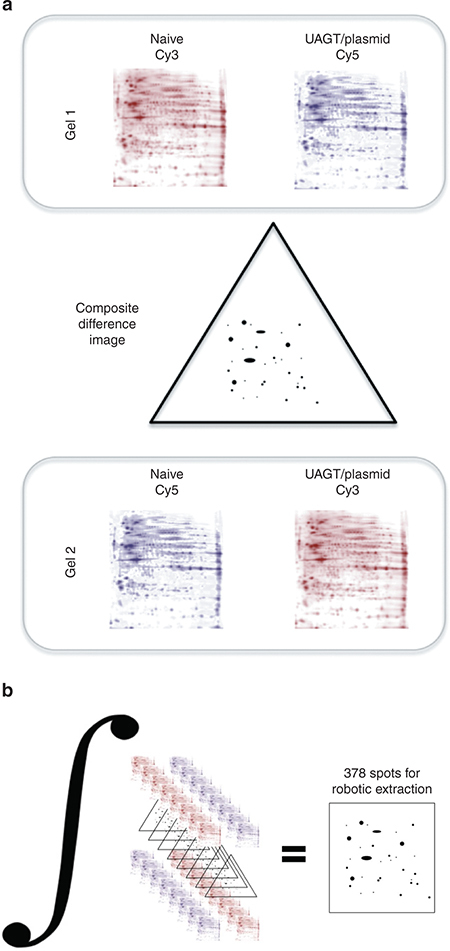
Diagram of our bioinformatic workflow. (**a**) Comparison of two randomly paired samples from the two groups to be compared (*e.g.*, naive and ultrasound-assisted gene transfer/plasmid) is carried out by labeling one sample with Cy3 and the other sample with Cy5 and running on the same gel to obtain a difference image. Cy dyes are then swapped and a second get is run to correct for Cy dye intensity differences. (**b**) The step shown in (**a**) is repeated for each randomly paired sample set and the difference images are integrated across all eight sample pairings. Analysis of variance is performed on a spot-by-spot basis to arrive at a final dataset of protein spots significantly different between the two groups.

**Figure 5:**
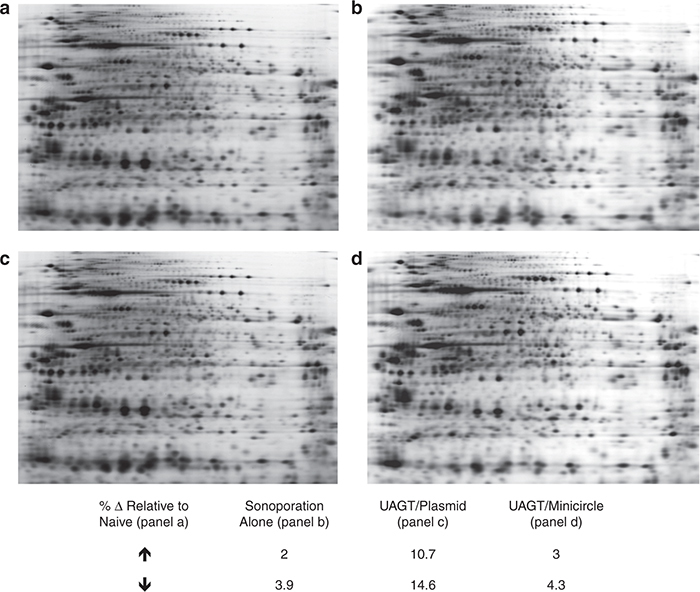
Composite proteomic profiles of salivary glands following gene transfer with nonviral vectors. (**a**) naive salivary gland, (**b**) salivary gland 24 hours following sonoporation in the absence of a plasmid vector, (**c**) salivary gland 24 hours following sonoporation in the presence of pCMV-GL3enh plasmid, (**d**) and salivary gland 24 hours following sonoporation in the presence of the CMV-GL3enh minicircle. Calculated differences as determined by DeCyder analysis (threshold = 2, *P* value = <0.05) are presented in the table, with each experimental condition being compared to naive.

The mechanism underlying UAGT is thought to occur primarily through the formation of transient pores in the cell membrane created by the inertial cavitation that occurs when microbubbles implode in the presence of an acoustic field of the appropriate frequency and power.^15–18^ While not fatal to cells under optimal conditions, we assumed that the biophysical insult resulting from sonoporation would elicit a physiological response within target cells that would be reflected in the proteomic profile. Using gel-based proteomic profiling (see Figure 4 for diagram of workflow), we sought to measure the magnitude of this response for later comparison with UAGT. Figure 5b shows the composite proteomic fingerprint of a mouse salivary gland, created by the DeCyder software (GE Healthcare, Piscataway, NJ) by integrating eight salivary gland samples, taken from mice 24 hours after sonoporation alone, versus a composite proteome of a naive gland integrated across six samples. To our surprise, sonoporation alone exerted only minor effects (>6%) upon the proteomic profile of the gland using our standard thresholds.

### Proteomic profiling of salivary gland following UAGT reveals major alterations in the proteomic profile that are attributable to the plasmid backbone

We have previously shown that adeno-associated virus vector-mediated gene transfer to the salivary gland results in profound alterations of the salivary gland proteome in the absence of histological manifestations of inflammation or tissue damage.^19^ After finding that sonoporation alone had little effect upon the proteome of the salivary gland, we wondered whether the addition of a plasmid vector to sonoporation (*i.e.*, UAGT) would have a detectable effect on the proteome. Figure 5c shows results of this experiment. We found >25% of all proteins were altered 24 hours after UAGT relative to naive gland. These results indicate that plasmid-mediated gene transfer exerts a profound effect upon the mouse salivary gland, approaching in magnitude that seen with virus-mediated gene transfer.

### UAGT with minicircles eliminates >95% of the proteomic alterations associated with first-generation plasmids

Based upon these results, we could not be certain whether these effects were due to noneukaryotic sequences in the plasmid backbone, generalized effects of foreign gene transfer, or a combination of the two. In order to parse these potential contributions to the observed phenomenon, we performed UAGT dose of minicircle vector equimolar to that of plasmid used in the experiment reported in Figure 5c. The minicircle contained an expression cassette identical in all respects to the first-generation plasmid. Figure 5d shows results of these experiments, demonstrating that the minicircle construct obviates the proteomic alterations seen with the first-generation plasmid, making it nearly indistinguishable from sonoporation alone.

### Proteins identified and pathway analysis

In order to make an initial exploration into the organ response to plasmid-mediated gene transfer, we picked 378 spots that were altered in plasmid versus naive but not altered in minicircle versus naive. Of these, 237 were conclusively identified using matrix-assisted laser desorption/ionization time-of-flight mass spectrometry, and their identities are listed in Table 1. Briefly, protein identification by peptide mass fingerprinting was done using the Bruker Daltonics FLEX series software includes several engines called flexAnalysis, BioTools, and Matrix Science MASCOT Search. The MASCOT search utility reports the highest probable hits with as a histogram of Mowse scores. Positive protein ID’s were reported only in those cases where one hit, and only one hit, achieved a Mowse score greater than 73. All proteins identified thusly were then brought back to BioTools and the MS spectrum was annotated with matched peptides as an additional quality control step. In cases where multiple hits exceeded a Mowse score of 73, those proteins were not reported. Table 1 therefore reflects proteins identified with the highest level of confidence, and 141 of the protein spots picked were omitted due to failure to meet this standard.

Using the GO-Elite pathway analysis tool,^20^ the proteins identified were compiled into 68 common interaction pathways, containing 2 or more identified proteins, and these are listed in Table 2. Visual output of the pathway analysis highlighting our proteins-of-interest are presented in full in the Supplementary Material. Of the 68 pathways implicated, 18 contained >3 of our proteins of interest, and several of these are reviewed in the discussion below.

## Discussion

UAGT to the salivary gland presents a unique model system in which to answer fundamental questions of nonviral vectorology. The salivary gland is an epithelium-derived, encapsulated structure that communicates with the oral cavity via the salivary duct. This anatomy facilitates delivery of gene transfer material via bloodless cannulation of the salivary duct and retrograde infusion. Because the contents of the intraductal labyrinth of the salivary gland can be controlled by external manipulation, we are able to precisely control the gene transfer conditions. This in turn allows us to parse out the effects on organ physiology of various viral and nonviral vectors using sonoporation. We previously utilized this technique to examine the effect of adeno-associated virus-mediated gene transfer on the mouse salivary gland proteome^19^ and documented profound global proteomic alterations that were dose dependent, even in the absence of extracellular inflammatory host response.

We were initially surprised to find that sonoporation alone, without the addition of a gene transfer vector, had minimal effects upon the proteome of the salivary gland. We had assumed that the inertial cavitation phenomenon underlying sonoporation would have substantial short-term effects upon membrane physiology and possibly structural proteins, even though we have documented that UAGT does not cause overt histological damage to the salivary gland. Nevertheless, our observation that sonoporation alone does not substantially alter the proteome presents a unique opportunity to study organ responses to various nonviral vectors, specifically first-generation plasmids and minicircles, in isolation from other confounding factors.

The limitations of gel-based proteomic profiling have been described^11^ and include the following: (i) failure to visualize high pI proteins, (ii) a focus on magnitude of change in spot intensity, which will not detect small but potentially important changes in proteins such as phosphorylation of G proteins, and (iii) inability to visualize hydrophobic (*e.g.*, membrane-associated) or highly glycosylated proteins. Thus, our results are best viewed as the selected pieces of a much larger puzzle that implicate some pathways in the host response to plasmid vectors but may not detect all pathways of importance. These limitations notwithstanding, there is no doubt that these results accurately reflect the relative magnitude of proteomic alterations following plasmid versus minicircle gene transfer, and these results implicate the plasmid backbone, whether due to its size, sequence, or both, as a major stimulator of innate immunity even in the absence of viral antigens. It should be noted that while efforts were made to ensure that the plasmid and the minicircle vector were as isogenic as possible, with the sequence differing only in the backbone region, they were prepared in different *Escherichia coli* lines, raising the possibility that methylation patterns and secondary structure may differ between the two vectors.

In instances where our analysis revealed clustering of our identified proteins in common pathways, the convergence was upon innate immunity, including most prominently Type 2 interferon, tumor necrosis factor-α/NF-κB, and Wnt. Additional innate signaling pathways implicated were interleukins 2, 3, 4, 5, 6, and 7, as well as Toll-like receptor signaling. Our findings may be considered relative to an earlier study by Mann *et al.*^21^ that documented changes in gene expression 1, 4, 7, and 14 days following gene electrotransfer to muscle tissue. Mann *et al.* also reported upregulation of Type 2 interferon and a number of interleukins (2, 6, and 12) following plasmid electrotransfer, a finding that is consistent with our results and may reflect the downstream gene expression consequences of the early proteomic changes that we have described. Notably, Mann *et al.* also reported that the effects of electroporation with a plasmid vector present were ~20-fold greater than electroporation alone, strikingly similar to our observations. The identities of proteins identified as being substantially altered as a result of UAGT with first-generation plasmid vectors, but not with minicircle vectors, present an interesting albeit incomplete picture of intracellular host response to nonviral vectors that highlights innate immune response and morphogenesis.

It should be appreciated that the present state of open-source pathway analysis in proteomics is extremely limited, with Wikipathways only coming online in 2008. In fact, of the 237 proteins (Table 1) identified as significantly altered between naive salivary gland and salivary gland following UAGT with a first-generated plasmid vector, only a few dozen were classified to known Wikipathways by the GO-ELITE analysis. Of these, a clear signaling cascade could be observed in only two cases, that of the Sos1-Hras1-Araf axis (leading to MEK/ERK activation)^22^ and the TRIF-Traf3-IKKepsilon axis (stimulated by Tlr3/4 and leading to Irf3 activation).^23^ This demonstrates in stark detail both the power and limitations of whole-proteome profiling. With respect to the former, we can generate “hits”, allowing us to focus on implicated pathways for more nuanced analysis of cell biology. With respect to the latter, the vast majority of our data cannot yet be reduced to a known pathway and thus teaches us relatively little beyond a quantitative measure of the extent of global proteomic alteration stimulated by the plasmid backbone.

If we accept the premise that gene transfer should have the most minimal collateral effects upon the target tissue as possible, then these results clearly demonstrate advantages of minicircle vectors as gene transfer agents in the salivary gland, both with regard to gene transfer efficiency and what might be termed “intracellular toxicity”. Our group is developing a nonviral approach to attempt to mimic positive reports of therapeutic efficacy in a human clinical trial utilizing adenovirus-mediated gene transfer of aquaporin-1 to the salivary glands of humans suffering from radiation-induced xerostomia.^24,25^ In this effort, the translational relevance of these findings should be directly relevant to the design of nonviral vectors for salivary gland gene therapy. Further, a number of clinical trials involving plasmid-mediated gene transfer to such tissues as skin, muscle, and heart have shown promising results.^26–29^ It remains to be seen whether other organs or tissues show a similar intracellular reaction to plasmid vectors, but this seems likely to be the case, as Mann *et al.*^21^ suggest. Going forward, the clinical importance of these intracellular responses must be evaluated, particularly in applications requiring episodic readministration of plasmid-mediated gene transfer. The assumption that plasmid-mediated gene transfer has minimal impact upon the host immune response should be reconsidered, and a minimalist approach to vector construction (*e.g.*, minicircles or mini-intronic plasmids)^30^ is appealing not just with regard to expression duration but also with regard to potential tissue toxicity.

## Materials and Methods

### Animals

All animal studies were conducted at Allegheny General Hospital, Pittsburgh, PA and were approved by the Institutional Animal Care and Use Committee. Wild-type C57/BL6 animals were bred at Allegheny General Hospital and male mice were used for all studies.

### Construction and preparation of vectors

The pGL3-enhancer vector was purchased from Promega (Madison, WI) and a canonical CMV promoter was inserted in the multiple cloning site. The entire expression cassette, including promoter, luciferase open reading frame, polyA, and enhancer were subcloned into the pAAV-MCS (Stratagene, La Jolla, CA) vector, resulting in pCMV-GL3enh. The expression cassette was then excised from pCMV-GL3enh and ligated into the pMC.BESPX-MCS1 vector (System Biosciences, Mountain View, CA; #MN100A-1), resulting in pMC-CMV-GL3enh (both vectors shown in Figure 1a). Full sequence information for both of these vectors is provided in the Supplementary Material. Minicircle construction was performed in the ZYCY10P3S2T bacterial strain per manufacturer’s instructions. Both vectors were prepared for experiments by purification using CsCl gradient centrifugation. Absorbance ratios were 1.89 (260/280) and 1.86 (260/230) for the plasmid and 1.98 (260/280) and 1.47 (260/230) for the minicircle. Endotoxin levels were measured in the final preps using a ToxinSensor kit (GenScript, Piscataway, NJ) according to manufacturer’s instructions. Endotoxin levels in the plasmid and minicircle were 0.082 and 0.041 EU/ml, respectively and did not differ substantially from a plasmid purified using a PureYield Plasmid Mini kit (0.097 EU/ml) including the endotoxin removal step (Promega, Madison, WI).

### UAGT to salivary glands

UAGT was performed as previously described.^8^ Briefly, the submandibular duct was cannulated bilaterally and a 50 µl solution containing 15% v/v Definity microbubbles and 1 g/l of plasmid vector (or the equimolar equivalent of minicircle vector) in phosphate-buffered saline (PBS) was infused. Bubbles were destroyed by 4 × 30 seconds bursts from a Sonigene device (Visualsonics, Toronto, ON, Canada) set for 1 MHz, 50% duty cycle, and 2 W/cm^2^, with 10 seconds between pulses. Following the four pulses, the emitter was withdrawn and the animal allowed to rest for 10 minutes before the catheter was removed.

### Bioluminescent imaging

Mice were anesthetized and injected intraperitoneally with the D-Luciferin substrate (Caliper Life Sciences, Hopkinton, MA) at a dose of 150 mg/10 g body weight. The mice were then placed in the IVIS Lumina II chamber containing a cryogenically cooled charge-coupled device camera to quantify photons spontaneously emitted by the animal. A standardized area-of-interest centered on the salivary gland was applied and total flux within this area was quantified as the average photons emitted/second/cm^2^ over a 60-second sampling period.

### Histological analysis

A cohort of animals (*n* = 6) underwent UAGT with a plasmid encoding human α-1-antitrypsin and were sacrificed 48 hours later and salivary gland removed and fixed in 5% paraformaldehyde in PBS pH 7.4 overnight. After processing for paraffin embedding, slices of 5 µm were cut per sample. The tissues were then deparaffinized and rehydrated. Hydrated tissues were subjected to antigen retrieval by incubating in 20 g/l proteinase K in buffer TE pH 8.0 at 37 °C during 10 minutes. The endogenous peroxidase was quenched for 10 minutes using 3% H_2_O_2_ in methanol followed by washing in PBS buffer pH 7.4. Prior to incubation with primary antibody, the sections were blocked with 10% fetal bovine serum, 04% saponin, 0.02% NaN_3_ in PBS buffer pH 7.4. A rabbit anti-A1AT polyclonal antibody (Dako, Glostrup, Denmark) diluted 1/800 in blocking buffer was incubated with the sections overnight at 4 °C. After a PBS wash step to remove unbound antibody, the detection of the primary antibody was realized using Histostain Plus 3^rd^ Gen IHC detection kit (Invitrogen, Carlsbad, CA). Before mounting in CitraMount medium, the nuclei were counterstained with Mayer’s hematoxylin. The slides were scanned in a VS120 microscope slide scanner (Olympus, Japan) and pictures were taken using an objective of ×20 and ×40. For the histopathological analysis, the deparaffinized sections were stained using hematoxylin/eosin following standard procedures.

### Proteomic profiling using 2-D difference gel electrophoresis

Twenty-four hours after UAGT, animals were sacrificed and salivary glands were harvested and homogenized in ice-cold T-PER (Pierce, Rockford, IL) with a protease inhibitor cocktail (Roche Diagnostics, Mannheim, Germany). Difference gel electrophoresis analysis was performed as previously described in detail.^19^ Technical details of difference gel electrophoresis analysis were duplicated exactly as described in this earlier report, including dye swapping and manual quality control of DeCyder output. Figure 4 graphically illustrates the workflow of the proteomic profiling technique.

### Image analysis

Gel analysis was performed using DeCyder DIA and BVA engines (GE Healthcare, Piscataway, NJ), a 2-D gel analysis software package designed specifically for the analysis of multiple difference gel electrophoresis experiments. This software package utilizes proprietary software to manage background subtraction, in-gel normalization, gel artifact removal, gel-to-gel matching, and statistical analysis. Manual input is restricted to setting threshold, *P* values, and manual spot checking as a final quality-control step to ensure that automated exclusion and inclusion of spots is appropriate. The estimated number of spots for each codetection procedure was set to 4,000. As recommended, an exclusion filter was applied to remove spots with a slope greater than one in order to reject spots that were likely to be contaminated with dust particles. A fixed value of 2.0 (±2) was used as the threshold to determine differentially expressed proteins, and *P* values were set at <0.05 to determine statistically significant differences between groups.

### Protein spot extraction and identification

Based upon DeCyder analysis, 378 spots were chosen for extraction from the gels using an Ettan Spot Piker automated robot arm. This device integrates with the DeCyder software and allows precise extraction of spots-of-interest without manual input. Extracted spots were then in-gel digested. Briefly, gel slices (1.4 mm in diameter) were soaked in 200 µl of 25 mmol/l ammonium bicarbonate and incubated at room temperature for 15 minutes. The supernatant was removed and discarded and this step was repeated three times. Next, gel slices were immersed in 200 µl 100% acetonitrile and incubated at room temperature for 30 seconds. The supernatant was removed and discarded. Remaining liquid in the gel pieces was removed by SpeedVac for 5 minutes and then rehydrated in trypsin-containing solution (Promega; #V5280) for 20 minutes at a concentration of 20 ng/ml and then covered above with 25 mmol/l ammonium bicarbonate. Protein was digested at 37 °C overnight (16–18 hours). Tryptic peptides were extracted from gel slices using 0.2 µl C18 resin ZipTip procedure (EMD Millipore, Billerica, MA; #ZTC18M960) according to manufacturer’s protocol. Final extract was eluted with TA30 solvent (30:70 [v/v] acetonitrile: 0.1% trifluoroacetic acid in water) containing a-cyano-4-hydroxycinnamic acid at 10 mg/ml (Bruker Daltonik GmbH, Bremen, Germany; #255344) and applied on ground steel matrix-assisted laser desorption target plate. A Bruker matrix-assisted laser desorption/ionization time-of-flight ultraflextreme mass spectrometer was used to characterize the protein fingerprint spectra. Spectra were queried on an in-house MASCOT server to determine protein identities, and estimated pI and molecular weight were determined from gels and considered in positively identifying proteins where spectra alone identified multiple identity candidates.

### Pathway and protein function analysis

GO-Elite (http://www.genmapp.org/go_elite) was used for the integration of identified proteins with biomolecular interaction networks (WikiPathways).^31^ All calculations and visual integration of the network with expression profiles (the final gene list) was done with probe sets at *P* < 0.05. For the interpretation, crosschecking, and visualization of the data, we also used AmiGO Term Enrichment and GO Slimmer (http://www.geneontology.org), GO Term Finder Cluster, and Tree View to perform cluster analysis using Euclidean distance algorithms of log-transformed, normalized raw data.

## Figures and Tables

**Table 1:** Identified proteins

*Protein name*	*Accession*
1-phosphatidylinositol 4,5-bisphosphate phosphodiesterase Δ-3	PLCD3_MOUSE
2′-5′-oligoadenylate synthase 3	OAS3_MOUSE
26S protease regulatory subunit 8	PRS8_MOUSE
2-amino-3-carboxymuconate-6-semialdehyde decarboxylase	ACMSD_MOUSE
6-phosphofructo-2-kinase/fructose-2,6-bisphosphatase 2	F262_MOUSE
72 kDa inositol polyphosphate 5-phosphatase	INP5E_MOUSE
A disintegrin and metalloproteinase with thrombospondin motifs 4	ATS4_MOUSE
Abhydrolase domain-containing protein FAM108C1	F108C_MOUSE
Actin-binding Rho-activating protain	ABRA_MOUSE
Actin-related protein T2	ACTT2_MOUSE
Activator of apoptosis harakiri	HRK_MOUSE
Acylphosphatase-1	ACYP1_MOUSE
Adenylsuccinate synthetase isozyme 1	PURA1_MOUSE
ADP-ribosylation factor-like protein 4D	ARL4D_MOUSE
AF4/FMR2 family member 1	AFF1_MOUSE
Alcohol dehydrogenase [NADP(+)]	AK1A1_MOUSE
Aldehyde dehydrogenase family 3 member B1	AL3B1_MOUSE
α-N-acetylgalactosaminidase	NAGAB_MOUSE
Alsin	ALS2_MOUSE
AN1-type zinc finger protein 1	ZFAN1_MOUSE
Angiogenin	ANGI_MOUSE
Angiopoietin-2	ANGP2_MOUSE
Angiopoietin-related protein	ANGL1_MOUSE
Ankyrin repeat domain-containing protein 24	ANR24_MOUSE
Aspartate-tRNA ligase cytoplasmic	SYDC_MOUSE
Ataxin-7	ATX7_MOUSE
ATP-binding cassette subfamily B member 8	ABCB8_MOUSE
Autophagy-related protein 2 homolog B	ATG2B_MOUSE
Bcl10-interacting CARD protein	BINCA_MOUSE
Bcl-2 homologous antagonist/killer	BAK_MOUSE
BEN domain-containing protein 3	BEND3_MOUSE
β-1,4 N-acetylgalactosan-minyltransferase 1	B4GN1_MOUSE
β-1-syntrophin	SNTB1_MOUSE
BTB/POZ domain containing protein KCTD11	KCD11_MOUSE
Calcium uptake protein 1, mitochondrial	MICU1_MOUSE
Calcium-transporting ATPase Type 2C member 1	AT2C1_MOUSE
Calpain-7	CAN7_MOUSE
cAMP-specific 3′,5′-cyclic phosphodiesterase 4A	PDE4A_MOUSE
CAP-Gly domain-containing linker protein 1	CLIP1_MOUSE
Casein kinase II subunit α	CSK22_MOUSE
CB1 cannabinoid receptor-interaction protein 1	CNRP1_MOUSE
CD48 antigen	CD48_MOUSE
Centrisomal protein of 170 kDa protein B	C170B_MOUSE
Cholesterol 7-α-monooxygenase	CP7A1_MOUSE
Choline dehydrogenase, mitochondrial	CHDH_MOUSE
Coatomer subunit β	COPB2_MOUSE
Coiled-coil domain-containing protein 148	CC148_MOUSE
Coiled-coil domain-containing protein 164	CC164_MOUSE
COMM domain-containing protein 9	COMD9_MOUSE
COP9 signalosome complex subunit 4	CSN4_MOUSE
C-type lectin domain family 2 member I	CLC2I_MOUSE
Cystatin-C	CYTC_MOUSE
Cytochrome b-c1 complex subunit 9	QCR9_MOUSE
Cytochrome p450	CP1A2_MOUSE
Cytochrome P450 2C50	CY250_MOUSE
Cytoplasmic dynein 1 light intermediate chain 1	DC1L1_MOUSE
Cytoplasmic dynein 2 heavy chain 1	DYHC2_MOUSE
DCC-interacting protein 13-β	DP13B_MOUSE
Disintegrin and metalloproteinase domain-containing protein 15	ADA15_MOUSE
DNA replication licensinf factor MCM7	MCM7_MOUSE
DnaJ homolog subfamily C member 28	DJC28_MOUSE
Docking protein 1	DOK1_MOUSE
Docking protein 6	DOK6_MOUSE
Dual specificity mitogen-activated protein kinase 4	MP2K4_MOUSE
Dynamin-1-like protein	DNM1L_MOUSE
E3 SUMO-protein ligase PIAS4	PIAS4_MOUSE
E3 ubiquitin-protein ligase MARCH3	MARH3_MOUSE
E3 ubiquitin-protein ligase RNF169	RN169_MOUSE
Echinoderm microtubule-associated protein-like 4	EMAL4_MOUSE
Ecto-ADP-ribosyltransferase 5	NAR5_MOUSE
Ectoderm-neural cortex protein 2	ENC2_MOUSE
Ecto-NOX disulfide-thiol exchanger 2	ENOX2_MOUSE
Electron transfer flavoprotein subunit α, mitochondrial	ETFA_MOUSE
Ephrin type-B receptor 1	EPHB1_MOUSE
Ethanolamine-phosphate cytidylyltransferase	PCY2_MOUSE
Exophilin-5	EXPH5_MOUSE
Fibronectin Type 3 and ankyrin repeat domains 1 protein	FANK1_MOUSE
Flotillin-2	FLOT2_MOUSE
Fragile X mental retardation syndrome-related protein 1	FXR1_CRIGR
Fragile X mental retardation syndrome-related protein 2	FXR2_MOUSE
G patch domain-containing protein 2	GPTC2_MOUSE
γ-crystallin A	CRGA_MOUSE
GAS2-like protein 2	GA2L2_MOUSE
GDNF-inducible zinc finger protein 1	GZF1_MOUSE
General transcription factor 3C polypeptide 4	TF3C4_MOUSE
Glial fibrillary acidic protein	GFAP_MOUSE
Glyceraldehyde-3-phosphate dehydrogenase	G3P_MOUSE
Glycine receptor subunit α-4	GLRA4_MOUSE
Glycogen phosphorylase	PYGM_MOUSE
Glyoxalase domain-containing protein 5	GLOD5_MOUSE
Golgi SNAP receptor complex member 1	GOSR1_MOUSE
Golgin subfamily A member 3	GOGA3_MOUSE
GRB2-associated and regulator of MAPK protein-like	GAREL_MOUSE
GS homeobox 1	GSX1_MOUSE
GTPase Hras	RASH_MOUSE
Guanine nucleotide-binding protein subunit β-2-like 1	GBLP_MOUSE
Heat shock cognate 71 kDa protein	HSP7C_MOUSE
Hemojuvelin	RGMC_MOUSE
Huntingtin-interacting protein 1-related protein	HIP1R_MOUSE
Hydrocephalus-inducing protein	HYDIN_MOUSE
Ig heavy chain V region J558	HVM13_MOUSE
Influenza virus NS1BP-binding protein homolog	NS1BP_MOUSE
Inhibitor of nuclear factor κ-B kinase subunit epsilon	IKKE_MOUSE
Initiation factor 4A-III	IF4A3_MOUSE
Integrator complex subunit 6	INT6_MOUSE
Integrin β-2	ITB2_MOUSE
Interleukin-22	IL22_MOUSE
Interleukin-22b	IL22B_MOUSE
Intraflagellar transport protein 57	IFT57_MOUSE
Intraflagellar transport protein 74 homolog	IFT74_MOUSE
Kelch-like protein 36	KLH36_MOUSE
Kinesin-like protein KLP6	KLP6_MOUSE
Kinocilin	KNCN_MOUSE
Laminin subunit α-5	LAMA5_MOUSE
Lens epithelial cell protein LEP503	LENEP_MOUSE
Leucine-rich repeat-containig protein 23	LRC23_MOUSE
Leucine-rich repeat-containing protein 7	LRRC7_MOUSE
Long-chain-fatty-acid-CoA ligase	ACSL6_MOUSE
Macoilin	MACOI_MOUSE
MAGE-like protein 2	MAGL2_MOUSE
MAGUK p55 subfamily member 2	MPP2_MOUSE
Megakaryocyte-associated tyrosine-protein kinase	MATK_MOUSE
Methylmalonate-semialdehyde dehydrogenase [acylating], mitochondrial	MMSA_MOUSE
Microphage scavenger receptor Types 1 and 2	MSRE_MOUSE
Mitogen-activated kinase	M3KL4_MOUSE
Mitotic-spindle organizing protein 2	MZT2_MOUSE
MORN repeat-containing protein 4	MORN4_MOUSE
Muscular LMNA-interactiong protein	MLIP_MOUSE
Myoferlin	MYOF_MOUSE
Myosin-1	MYH1_MOUSE
Myosin-4	MYH4_MOUSE
Myotubularin	MTM1_MOUSE
NACHT, LRR and PYD daomains-containing protein 5	NALP5_MOUSE
Nephronectin	NPNT_MOUSE
Neutrophil cytosol factor 1	NCF1_MOUSE
Nuclear pore complex protein Nup85	NUP85_MOUSE
Nucleolar GTP-binding protein 1	NOG1_MOUSE
Nucleolar protein 16	NOP16_MOUSE
Nucleolar transcription factor 1	UBF1_MOUSE
Opalin	OPALI_MOUSE
Pantothenate kinase 4	PANK4_MOUSE
Paraspeckle component 1	PSPC1_MOUSE
Peptidyl-prolyl cis-trans isomerase FKBP5	FKBP5_MOUSE
Peroxiredoxin-1	PRDX1_MOUSE
Phenylalanine-4-hydroxylase	PH4H_MOUSE
Phosphate carrier protein mitochondrial	MPCP_MOUSE
Phosphatidylinositol 3-kinase catalytic subunit Type 3	PK3C3_MOUSE
Phosphatidylinositol transfer protein β isoform	PIPNB_MOUSE
Phosphorylated CTD-interacting factor 1	PCIF1_MOUSE
Plexin-B2	PLXB2_MOUSE
Poly (a) polymerase γ	PAPOG_MOUSE
Poly(U)-specific endoribonuclease	ENDOU_MOUSE
Prickle-like protein	PRIC1_MOUSE
Probable ATP-dependent RNA helicase DDX6	DDX6_MOUSE
Programmed cell death protein 4	PDCD4_MOUSE
Prolactin-7D1	PR7D1_MOUSE
Protein Asterix	ASTER_MOUSE
Protein Daple	DAPLE_MOUSE
Protein FAM216B	F216B_MOUSE
Protein FAM229B	F229B_MOUSE
Protein kinase C delta-binding protein	PRDBP_MOUSE
Protein naked cuticle homolog 1	NKD1_MOUSE
Protein N-terminal asparagine amidohydrolase	NTAN1_MOUSE
Protein RER1	RER1_MOUSE
Protein TCL1B1	TCLB1_MOUSE
Putative ATP-dependent RNA helicase TDRD9	TDRD9_MOUSE
Putative polycomb group protein ASXL2	ASXL2_MOUSE
Pyridoxal-dependent decarboxylase domain-containing protein 1	PDXD1_MOUSE
Pyroglutamyl-peptidase 1-like protein	PGPIL_MOUSE
Pyruvate dehydrogenase (acetyl-transferring)-phosphatase 1	PDP1_MOUSE
Pyruvate dehydrogenase (lipoamide) kinase isozyme 2 mitochondrial	PDK2_MOUSE
Pyruvate dehydrogenase (lipoamide) kinase isozyme 3 mitochondrial	PDK3_MOUSE
Rap guanine nucleotide exchange factor 5	RPGF5_MOUSE
Ras-related protein Rab-19	RAB19_MOUSE
Receptor-transporting protein 4	RTP4_MOUSE
Receptor-type tyrosine-protein phosphatase U	PTPRU_MOUSE
Regulator of G-protein signaling 4	RGS4_MOUSE
Rho GTPase-activating protein 1	RHG01_MOUSE
Sarcolemmal membrane-associated protein	SLMAP_MOUSE
Semaphorin-3A	SEM3A_MOUSE
Semaphorin-7A	SEM7A_MOUSE
Seminal vesicle secretory protein 5	SVS5_MOUSE
Septin-2	SEPT2_MOUSE
Septin-8	SEPT8_MOUSE
Serine/threonine-protein kinase ICK	ICK_MOUSE
Serine/threonine-protein kinase Nek11	NEK11_MOUSE
Serine/threonine-protein kinase Nek5	NEK5_MOUSE
Serine/threonine-protein kinase PLK4	PLK4_MOUSE
Serine/threonine-protein kinase SMG1	SMG1_MOUSE
Serpin B12	SPB12_MOUSE
Serpin H1	SERPH_MOUSE
Serum albumin	ALBU_MOUSE
Small nuclear ribonucleoprotein-associated protein N	RSMN_MOUSE
Son of sevenless homolog 1	SOS1_MOUSE
Spermatogenesis-associated protein 7 homolog	SPAT7_MOUSE
SRC kinase signaling inhibitor 1	SRCN1_MOUSE
Sulfite oxidase, mitochondrial	SUOX_MOUSE
SUN domain-containing protein 1	SUN1_MOUSE
Synaptotagmin-6	SYT6_MOUSE
T-box transcription factor TBX21	TBX21_MOUSE
Testin	TES_MOUSE
TIR domain-containing adapter molecule 1	TCAM1_MOUSE
Torsin-1B	TOR1B_MOUSE
Trafficing protein particle complex subunit 3-like protein	TPC3L_MOUSE
TRAF-interacting protein	TRAIP_MOUSE
Trans-2-enoyl-CoA reductase mitochondrial	MECR_MOUSE
Transketolase-like protein 2	TKTL2_MOUSE
Translocase of inner mitochondrial membrane domain-containing protein 1	TIDC1_MOUSE
tRNA guanine(26)-N(2)-dimethyltransferase	TRM1_MOUSE
Tubulin β-2B chain	TBB2B_MOUSE
Tudor domain-containing protein 3	TDRD3_MOUSE
Tumor protein p63-regulated gene 1 protein	TPRG1_MOUSE
Tyrosine-protein kinase ABL1	ABL1_MOUSE
Tyrosine-protein kinase ZAP-70	ZAP70_MOUSE
Tyrosine-protein phosphatase non-receptor Type 12	PTN12_MOUSE
Tyrosine-protein phosphatase non-receptor Type 13	PTN13_MOUSE
U7 snRNA-associated Sm-like protein LSm11	LSM11_MOUSE
Ubiquitin carboxyl-terminal hydrolase 13	UBP13_MOUSE
Ubiquitin carboxyl-terminal hydrolase 36	UBP36_MOUSE
UBX domain-containing protein	UBXN8_MOUSE
UDP-glucuronic acid decarboxylase	UXS1_MOUSE
UDP-N-acetylglucosamine-peptide N-acetylglucosaminyltransferase 110kDa subunit	OGT1_MOUSE
Uncharacterized protein C11orf89 homolog	CK089_MOUSE
Uncharacterized protein C2orf47 homolog	CB047_MOUSE
Uncharacterized protein C9orf114 homolog	CI114_MOUSE
Uncharacterized protein C9orf172 homolog	CI172_MOUSE
Upoplakin-1b	UPK1B_MOUSE
UV-stimulated scaffold protein A	UVSSA_MOUSE
Vacuolar fusion protein MON1 homolog B	MON1B_MOUSE
Vomeronasal Type 2 receptor 1	V2R1_MOUSE
V-set and immuniglobulin domain-containing protein 8	VSIG8_MOUSE
V-type proton ATPase subunit B	VATB2_MOUSE
WAP four-disulfide core domain protein 6B	WFC6B_MOUSE
WD repeat-containig protein 93	WDR93_MOUSE
Xin actin-binding repeat-containing protein2	XIRP2_MOUSE
Zinc finger protein 182	ZN182_MOUSE
Zinc finger protein 90	ZEP90_MOUSE

**Table 2:** Pathways identified

WikiPathway ID	3pp Specification	Number of protein(s)
WP310	mRNA processing	12
WP246	TNF-α NF-κB signaling pathway	7
WP1763	PluriNetWork	7
WP407	Kit receptor signaling pathway	6
WP572	EGFR1 signaling pathway	6
WP6	Integrin-mediated cell adhesion	5
WP65	Insulin signaling	5
WP539	Wnt signaling pathway NetPath	5
WP1253	Type 2 interferon signaling (IFNG)	5
WP85	Focal adhesion	4
WP251	MAPK cascade	4
WP274	B cell receptor signaling pathway	4
WP373	IL-3 signaling pathway	4
WP431	Nuclear receptors in lipid metabolism and toxicity	4
WP480	T-cell receptor signaling pathway	4
WP1261	ErbB signaling pathway	4
WP2087	miRNA regulation of DNA damage response	4
WP79	Tryptophan metabolism	3
WP190	Cell cycle	3
WP216	Striated muscle contraction	3
WP387	IL-6 signaling pathway	3
WP434	TCA cycle	3
WP488	α6-β4 integrin signaling pathway	3
WP493	MAPK signaling pathway	3
WP662	Amino acid metabolism	3
WP1251	Metapathway biotransformation	3
WP1254	Apoptosis	3
WP1262	Aflatoxin B1 metabolism	3
WP1267	Senescence and autophagy	3
WP1271	Toll-like receptor signaling pathway:KEGG	3
WP1983	Splicing factor NOVA-regulated synpatic proteins	3
WP2185	Purine metabolism:KEGG-mmu00230	3
WP2292	Chemokine signaling pathway:KEGG-mmu04062	3
WP88	Toll-like receptor signaling	2
WP93	IL-4 signaling pathway	2
WP151	IL-5 signaling pathway	2
WP163	Cytoplasmic ribosomal proteins	2
WP193	Signaling of hepatocyte growth factor receptor	2
WP232	G Protein signaling pathways	2
WP240	Alanine and aspartate metabolism	2
WP258	TGF-β receptor signaling pathway	2
WP297	IL-7 signaling pathway	2
WP336	Fatty acid biosynthesis	2
WP339	ESC pluripotency pathways	2
WP350	p38 MAPK signaling pathway (BioCarta)	2
WP413	G1 to S cell cycle control	2
WP447	Adipogenesis	2
WP450	IL-2 signaling pathway	2
WP458	Inflammatory response pathway	2
WP519	Proteasome degradation	2
WP523	Regulation of actin cytoskeleton	2
WP544	Circadian exercise	2
WP571	FAS pathway and stress induction of HSP regulation	2
WP723	Wnt signaling pathway and pluripotency	2
WP730	Glutathione and one carbon metabolism	2
WP1244	Estrogen signalling	2
WP1249	EPO receptor signaling	2
WP1264	Estrogen metabolism	2
WP1270	Endochondral ossification	2
WP1274	Cytochrome P450	2
WP1496	Oxidative damage	2
WP1560	MicroRNAs in cardiomyocyte hypertrophy	2
WP1770	One carbon metabolism and related pathways	2
WP2074	Neural crest differentiation	2
WP2310	PodNet: protein-protein interactions in the podocyte	2
WP2316	PPAR signaling pathway:KEGG-mmu03320	2
WP2432	Spinal cord injury	2

## References

[bib1] Nathwani AC Tuddenham EG Rangarajan S Rosales C McIntosh J Linch DC 2011 Adenovirus-associated virus vector-mediated gene transfer in hemophilia B N Engl J Med 365 2357 2365 22149959 10.1056/NEJMoa1108046 PMC3265081

[bib2] Miller JW 2008 Preliminary results of gene therapy for retinal degeneration N Engl J Med 358 2282 2284 18441372 10.1056/NEJMe0803081

[bib3] Hacein-Bey-Abina S Hauer J Lim A Picard C Wang GP Berry CC 2010 Efficacy of gene therapy for X-linked severe combined immunodeficiency N Engl J Med 363 355 364 20660403 10.1056/NEJMoa1000164 PMC2957288

[bib4] Shayakhmetov DM Di Paolo NC Mossman KL 2010 Recognition of virus infection and innate host responses to viral gene therapy vectors Mol Ther 18 1422 1429 20551916 10.1038/mt.2010.124 PMC2927067

[bib5] Muruve DA 2004 The innate immune response to adenovirus vectors Hum Gene Ther 15 1157 1166 15684693 10.1089/hum.2004.15.1157

[bib6] Zaiss AK Liu Q Bowen GP Wong NC Bartlett JS Muruve DA 2002 Differential activation of innate immune responses by adenovirus and adeno-associated virus vectors J Virol 76 4580 4590 11932423 10.1128/JVI.76.9.4580-4590.2002 PMC155101

[bib7] Vandermeulen G Marie C Scherman D Préat V 2011 New generation of plasmid backbones devoid of antibiotic resistance marker for gene therapy trials Mol Ther 19 1942 1949 21878901 10.1038/mt.2011.182 PMC3222533

[bib8] Passineau MJ Zourelias L Machen L Edwards PC Benza RL 2010 Ultrasound-assisted non-viral gene transfer to the salivary glands Gene Ther 17 1318 1324 20508599 10.1038/gt.2010.86

[bib9] Bao S Thrall BD Miller DL 1997 Transfection of a reporter plasmid into cultured cells by sonoporation *in vitro* Ultrasound Med Biol 23 953 959 9300999 10.1016/s0301-5629(97)00025-2

[bib10] Mastrangeli A O’Connell B Aladib W Fox PC Baum BJ Crystal RG 1994 Direct *in vivo* adenovirus-mediated gene transfer to salivary glands Am J Physiol 266 6 Pt 1 G1146 G1155 8023944 10.1152/ajpgi.1994.266.6.G1146

[bib11] Rabilloud T Chevallet M Luche S Lelong C 2010 Two-dimensional gel electrophoresis in proteomics: Past, present and future J Proteomics 73 2064 2077 20685252 10.1016/j.jprot.2010.05.016

[bib12] Chen ZY He CY Ehrhardt A Kay MA 2003 Minicircle DNA vectors devoid of bacterial DNA result in persistent and high-level transgene expression in vivo Mol Ther 8 495 500 12946323 10.1016/s1525-0016(03)00168-0

[bib13] Kay MA He CY Chen ZY 2010 A robust system for production of minicircle DNA vectors Nat Biotechnol 28 1287 1289 21102455 10.1038/nbt.1708 PMC4144359

[bib14] Chabot S Orio J Schmeer M Schleef M Golzio M Teissié J 2013 Minicircle DNA electrotransfer for efficient tissue-targeted gene delivery Gene Ther 20 62 68 22257936 10.1038/gt.2011.215

[bib15] Deng CX Sieling F Pan H Cui J 2004 Ultrasound-induced cell membrane porosity Ultrasound Med Biol 30 519 526 15121254 10.1016/j.ultrasmedbio.2004.01.005

[bib16] Zhou Y Kumon RE Cui J Deng CX 2009 The size of sonoporation pores on the cell membrane Ultrasound Med Biol 35 1756 1760 19647924 10.1016/j.ultrasmedbio.2009.05.012 PMC2752487

[bib17] Qiu Y Luo Y Zhang Y Cui W Zhang D Wu J 2010 The correlation between acoustic cavitation and sonoporation involved in ultrasound-mediated DNA transfection with polyethylenimine (PEI) *in vitro* J Control Release 145 40 48 20398711 10.1016/j.jconrel.2010.04.010

[bib18] Zhou Y Yang K Cui J Ye JY Deng CX 2012 Controlled permeation of cell membrane by single bubble acoustic cavitation J Control Release 157 103 111 21945682 10.1016/j.jconrel.2011.09.068 PMC3258473

[bib19] Geguchadze RN Machen L Zourelias L Gallo PH Passineau MJ 2012 An AAV2/5 vector enhances safety of gene transfer to the mouse salivary gland J Dent Res 91 382 386 22307036 10.1177/0022034512437373 PMC3310756

[bib20] Zambon AC Gaj S Ho I Hanspers K Vranizan K Evelo CT 2012 GO-Elite: a flexible solution for pathway and ontology over-representation Bioinformatics 28 2209 2210 22743224 10.1093/bioinformatics/bts366 PMC3413395

[bib21] Mann CJ Anguela XM Montané J Obach M Roca C Ruzo A 2012 Molecular signature of the immune and tissue response to non-coding plasmid DNA in skeletal muscle after electrotransfer Gene Ther 19 1177 1186 22170344 10.1038/gt.2011.198

[bib22] Roberts PJ Der CJ 2007 Targeting the Raf-MEK-ERK mitogen-activated protein kinase cascade for the treatment of cancer Oncogene 26 3291 3310 17496923 10.1038/sj.onc.1210422

[bib23] O’Neill LA Bowie AG 2007 The family of five: TIR-domain-containing adaptors in Toll-like receptor signalling Nat Rev Immunol 7 353 364 17457343 10.1038/nri2079

[bib24] Baum BJ Alevizos I Zheng C Cotrim AP Liu S McCullagh L 2012 Early responses to adenoviral-mediated transfer of the aquaporin-1 cDNA for radiation-induced salivary hypofunction Proc Natl Acad Sci USA 109 19403 19407 23129637 10.1073/pnas.1210662109 PMC3511089

[bib25] Baum BJ Zheng C Alevizos I Cotrim AP Liu S McCullagh L 2010 Development of a gene transfer-based treatment for radiation-induced salivary hypofunction Oral Oncol 46 4 8 19892587 10.1016/j.oraloncology.2009.09.004 PMC2818419

[bib26] Penn MS Mendelsohn FO Schaer GL Sherman W Farr M Pastore J 2013 An open-label dose escalation study to evaluate the safety of administration of nonviral stromal cell-derived factor-1 plasmid to treat symptomatic ischemic heart failure Circ Res 112 816 825 23429605 10.1161/CIRCRESAHA.111.300440

[bib27] Makino H Aoki M Hashiya N Yamasaki K Azuma J Sawa Y 2012 Long-term follow-up evaluation of results from clinical trial using hepatocyte growth factor gene to treat severe peripheral arterial disease Arterioscler Thromb Vasc Biol 32 2503 2509 22904270 10.1161/ATVBAHA.111.244632

[bib28] Vardas E Stanescu I Leinonen M Ellefsen K Pantaleo G Valtavaara M 2012 Indicators of therapeutic effect in FIT-06, a Phase II trial of a DNA vaccine, GTU(®)-Multi-HIVB, in untreated HIV-1 infected subjects Vaccine 30 4046 4054 22549090 10.1016/j.vaccine.2012.04.007

[bib29] Heller LC Heller R 2010 Electroporation gene therapy preclinical and clinical trials for melanoma Curr Gene Ther 10 312 317 20557286 10.2174/156652310791823489

[bib30] Lu J Zhang F Kay MA 2013 A mini-intronic plasmid (MIP): a novel robust transgene expression vector *in vivo* and *in vitro* Mol Ther 21 954 963 23459514 10.1038/mt.2013.33 PMC3666631

[bib31] Pico AR Kelder T van Iersel MP Hanspers K Conklin BR Evelo C 2008 WikiPathways: pathway editing for the people PLoS Biol 6 e184 18651794 10.1371/journal.pbio.0060184 PMC2475545

