# Supplementary material for: Proteomic profiling of salivary gland after nonviral gene transfer mediated by conventional plasmids and minicircles
Source: Mol Ther Methods Clin Dev. 2014 Apr 2;1:14007–. doi: 10.1038/mtm.2014.7 (PMC4236002; doi:10.1038/mtm.2014.7)
Supplement: Supplementary Material [file mtm20147-s1.doc]

pCMV-GL3enh

1 agcttggcat tccggtactg ttggtaaagc caccatggaa gacgccaaaa acataaagaa

61 aggcccggcg ccattctatc cgctggaaga tggaaccgct ggagagcaac tgcataaggc

121 tatgaagaga tacgccctgg ttcctggaac aattgctttt acagatgcac atatcgaggt

181 ggacatcact tacgctgagt acttcgaaat gtccgttcgg ttggcagaag ctatgaaacg

241 atatgggctg aatacaaatc acagaatcgt cgtatgcagt gaaaactctc ttcaattctt

301 tatgccggtg ttgggcgcgt tatttatcgg agttgcagtt gcgcccgcga acgacattta

361 taatgaacgt gaattgctca acagtatggg catttcgcag cctaccgtgg tgttcgtttc

421 caaaaagggg ttgcaaaaaa ttttgaacgt gcaaaaaaag ctcccaatca tccaaaaaat

481 tattatcatg gattctaaaa cggattacca gggatttcag tcgatgtaca cgttcgtcac

541 atctcatcta cctcccggtt ttaatgaata cgattttgtg ccagagtcct tcgataggga

601 caagacaatt gcactgatca tgaactcctc tggatctact ggtctgccta aaggtgtcgc

661 tctgcctcat agaactgcct gcgtgagatt ctcgcatgcc agagatccta tttttggcaa

721 tcaaatcatt ccggatactg cgattttaag tgttgttcca ttccatcacg gttttggaat

781 gtttactaca ctcggatatt tgatatgtgg atttcgagtc gtcttaatgt atagatttga

841 agaagagctg tttctgagga gccttcagga ttacaagatt caaagtgcgc tgctggtgcc

901 aaccctattc tccttcttcg ccaaaagcac tctgattgac aaatacgatt tatctaattt

961 acacgaaatt gcttctggtg gcgctcccct ctctaaggaa gtcggggaag cggttgccaa

1021 gaggttccat ctgccaggta tcaggcaagg atatgggctc actgagacta catcagctat

1081 tctgattaca cccgaggggg atgataaacc gggcgcggtc ggtaaagttg ttccattttt

1141 tgaagcgaag gttgtggatc tggataccgg gaaaacgctg ggcgttaatc aaagaggcga

1201 actgtgtgtg agaggtccta tgattatgtc cggttatgta aacaatccgg aagcgaccaa

1261 cgccttgatt gacaaggatg gatggctaca ttctggagac atagcttact gggacgaaga

1321 cgaacacttc ttcatcgttg accgcctgaa gtctctgatt aagtacaaag gctatcaggt

1381 ggctcccgct gaattggaat ccatcttgct ccaacacccc aacatcttcg acgcaggtgt

1441 cgcaggtctt cccgacgatg acgccggtga acttcccgcc gccgttgttg ttttggagca

1501 cggaaagacg atgacggaaa aagagatcgt ggattacgtc gccagtcaag taacaaccgc

1561 gaaaaagttg cgcggaggag ttgtgtttgt ggacgaagta ccgaaaggtc ttaccggaaa

1621 actcgacgca agaaaaatca gagagatcct cataaaggcc aagaagggcg gaaagatcgc

1681 cgtgtaattc tagagtcggg gcggccggcc gcttcgagca gacatgataa gatacattga

1741 tgagtttgga caaaccacaa ctagaatgca gtgaaaaaaa tgctttattt gtgaaatttg

1801 tgatgctatt gctttatttg taaccattat aagctgcaat aaacaagtta acaacaacaa

1861 ttgcattcat tttatgtttc aggttcaggg ggaggtgtgg gaggtttttt aaagcaagta

1921 aaacctctac aaatgtggta aaatcgataa ggatctgaac gatggagcgg agaatgggcg

1981 gaactgggcg gagttagggg cgggatgggc ggagttaggg gcgggactat ggttgctgac

2041 taattgagat gcatgctttg catacttctg cctgctgggg agcctgggga ctttccacac

2101 ctggttgctg actaattgag atgcatgctt tgcatacttc tgcctgctgg ggagcctggg

2161 gactttccac accctaactg acacacattc cacagcggat ccgtcgaccg atgcccttga

2221 gagccttcaa cccagtcagc tccttccggt gggcgcgggg catgactatc gtcgccgcac

2281 ttatgactgt cttctttatc atgcaactcg taggacaggt gccggcagcg ctgctcgaga

2341 gatctacggg tggcatccct gtgacccctc cccagtgcct ctcctggccc tggaagttgc

2401 cactccagtg cccaccagcc ttgtcctaat aaaattaagt tgcatcattt tgtctgacta

2461 ggtgtccttc tataatatta tggggtggag gggggtggta tggagcaagg ggcaagttgg

2521 gaagacaacc tgtagggcct gcggggtcta ttgggaacca agctggagtg cagtggcaca

2581 atcttggctc actgcaatct ccgcctcctg ggttcaagcg attctcctgc ctcagcctcc

2641 cgagttgttg ggattccagg catgcatgac caggctcagc taatttttgt ttttttggta

2701 gagacggggt ttcaccatat tggccaggct ggtctccaac tcctaatctc aggtgatcta

2761 cccaccttgg cctcccaaat tgctgggatt acaggcgtga accactgctc ccttccctgt

2821 ccttctgatt ttgtaggtaa ccacgtgcgg accgagcggc cgcaggaacc cctagtgatg

2881 gagttggcca ctccctctct gcgcgctcgc tcgctcactg aggccgggcg accaaaggtc

2941 gcccgacgcc cgggctttgc ccgggcggcc tcagtgagcg agcgagcgcg cagctgcctg

3001 caggggcgcc tgatgcggta ttttctcctt acgcatctgt gcggtatttc acaccgcata

3061 cgtcaaagca accatagtac gcgccctgta gcggcgcatt aagcgcggcg ggtgtggtgg

3121 ttacgcgcag cgtgaccgct acacttgcca gcgccctagc gcccgctcct ttcgctttct

3181 tcccttcctt tctcgccacg ttcgccggct ttccccgtca agctctaaat cgggggctcc

3241 ctttagggtt ccgatttagt gctttacggc acctcgaccc caaaaaactt gatttgggtg

3301 atggttcacg tagtgggcca tcgccctgat agacggtttt tcgccctttg acgttggagt

3361 ccacgttctt taatagtgga ctcttgttcc aaactggaac aacactcaac cctatctcgg

3421 gctattcttt tgatttataa gggattttgc cgatttcggc ctattggtta aaaaatgagc

3481 tgatttaaca aaaatttaac gcgaatttta acaaaatatt aacgtttaca attttatggt

3541 gcactctcag tacaatctgc tctgatgccg catagttaag ccagccccga cacccgccaa

3601 cacccgctga cgcgccctga cgggcttgtc tgctcccggc atccgcttac agacaagctg

3661 tgaccgtctc cgggagctgc atgtgtcaga ggttttcacc gtcatcaccg aaacgcgcga

3721 gacgaaaggg cctcgtgata cgcctatttt tataggttaa tgtcatgata ataatggttt

3781 cttagacgtc aggtggcact tttcggggaa atgtgcgcgg aacccctatt tgtttatttt

3841 tctaaataca ttcaaatatg tatccgctca tgagacaata accctgataa atgcttcaat

3901 aatattgaaa aaggaagagt atgagtattc aacatttccg tgtcgccctt attccctttt

3961 ttgcggcatt ttgccttcct gtttttgctc acccagaaac gctggtgaaa gtaaaagatg

4021 ctgaagatca gttgggtgca cgagtgggtt acatcgaact ggatctcaac agcggtaaga

4081 tccttgagag ttttcgcccc gaagaacgtt ttccaatgat gagcactttt aaagttctgc

4141 tatgtggcgc ggtattatcc cgtattgacg ccgggcaaga gcaactcggt cgccgcatac

4201 actattctca gaatgacttg gttgagtact caccagtcac agaaaagcat cttacggatg

4261 gcatgacagt aagagaatta tgcagtgctg ccataaccat gagtgataac actgcggcca

4321 acttacttct gacaacgatc ggaggaccga aggagctaac cgcttttttg cacaacatgg

4381 gggatcatgt aactcgcctt gatcgttggg aaccggagct gaatgaagcc ataccaaacg

4441 acgagcgtga caccacgatg cctgtagcaa tggcaacaac gttgcgcaaa ctattaactg

4501 gcgaactact tactctagct tcccggcaac aattaataga ctggatggag gcggataaag

4561 ttgcaggacc acttctgcgc tcggcccttc cggctggctg gtttattgct gataaatctg

4621 gagccggtga gcgtgggtct cgcggtatca ttgcagcact ggggccagat ggtaagccct

4681 cccgtatcgt agttatctac acgacgggga gtcaggcaac tatggatgaa cgaaatagac

4741 agatcgctga gataggtgcc tcactgatta agcattggta actgtcagac caagtttact

4801 catatatact ttagattgat ttaaaacttc atttttaatt taaaaggatc taggtgaaga

4861 tcctttttga taatctcatg accaaaatcc cttaacgtga gttttcgttc cactgagcgt

4921 cagaccccgt agaaaagatc aaaggatctt cttgagatcc tttttttctg cgcgtaatct

4981 gctgcttgca aacaaaaaaa ccaccgctac cagcggtggt ttgtttgccg gatcaagagc

5041 taccaactct ttttccgaag gtaactggct tcagcagagc gcagatacca aatactgtcc

5101 ttctagtgta gccgtagtta ggccaccact tcaagaactc tgtagcaccg cctacatacc

5161 tcgctctgct aatcctgtta ccagtggctg ctgccagtgg cgataagtcg tgtcttaccg

5221 ggttggactc aagacgatag ttaccggata aggcgcagcg gtcgggctga acggggggtt

5281 cgtgcacaca gcccagcttg gagcgaacga cctacaccga actgagatac ctacagcgtg

5341 agctatgaga aagcgccacg cttcccgaag ggagaaaggc ggacaggtat ccggtaagcg

5401 gcagggtcgg aacaggagag cgcacgaggg agcttccagg gggaaacgcc tggtatcttt

5461 atagtcctgt cgggtttcgc cacctctgac ttgagcgtcg atttttgtga tgctcgtcag

5521 gggggcggag cctatggaaa aacgccagca acgcggcctt tttacggttc ctggcctttt

5581 gctggccttt tgctcacatg tcctgcaggc agctgcgcgc tcgctcgctc actgaggccg

5641 cccgggcgtc gggcgacctt tggtcgcccg gcctcagtga gcgagcgagc gcgcagagag

5701 ggagtggcca actccatcac taggggttcc tgcggccgca cgcgtctagt tattaatagt

5761 aatcaattac ggggtcatta gttcatagcc catatatgga gttccgcgtt acataactta

5821 cggtaaatgg cccgcctggc tgaccgccca acgacccccg cccattgacg tcaataatga

5881 cgtatgttcc catagtaacg tcaataggga ctttccattg acgtcaatgg gtggagtatt

5941 tacggtaaac tgcccacttg gcagtacatc aagtgtatca tatgccaagt acgcccccta

6001 ttgacgtcaa tgacggtaaa tggcccgcct ggcattatgc ccagtacatg accttatggg

6061 actttcctac ttggcagtac atctacgtat tagtcatcgc tattaccatg gtgatgcggt

6121 tttggcagta catcaatggg cgtggatagc ggtttgactc acggggattt ccaagtctcc

6181 accccattga cgtcaatggg agtttgtttt gcaccaaaat caacgggact ttccaaaatg

6241 tcgtaacaac tccgccccat tgacgcaaat gggcggtagg cgtgtacggt gggaggtcta

6301 tataagcaga gctcgtttag tgaaccgtca gatcgcctgg agacgccatc cacgctgttt

6361 tgacctccat agaagacacc gggaccgatc cagcctccgc ggattcgaat cccggccggg

6421 aacggtgcat tggaacgcgg attccccgtg ccaagagtga cgtaagtacc gcctatagag

6481 tctataggcc cacaaaaaat gctttcttct tttaatatac ttttttgttt atcttatttc

6541 taatactttc cctaatctct ttctttcagg gcaataatga tacaatgtat catgcctctt

6601 tgcaccattc taaagaataa cagtgataat ttctgggtta aggcaatagc aatatttctg

6661 catataaata tttctgcata taaattgtaa ctgatgtaag aggtttcata ttgctaatag

6721 cagctacaat ccagctacca ttctgctttt attttatggt tgggataagg ctggattatt

6781 ctgagtccaa gctaggccct tttgctaatc atgttcatac ctcttatctt cctcccacag

6841 ctcctgggca acgtgctggt ctgtgtgctg gcccatcact ttggcaaaga attgggattc

6901 gaacatcgat tgaattcccc ggggatcctc tagagtcgac ctgcaga

pMC-CMV-GL3enh “Parental”

1 nnccctntta nccagatgac atnccnnntn tncctagatg acntaccnnt nncccagatg

61 acattaccng tatccctaga tacattaccc tgtntcccag atgacatacc tgttatccct

121 agatgacatt accngttatc ccagatgaca ttacctgtta tccctagata cattaccngt

181 tatcccagat gacataccct gttatccnta gatgacatta ccctgttatc ccagatgaca

241 ttaccctgtt atccctagat acattaccct gttatcccag atgacatacc ctgttatccc

301 tagatgacat taccctgtta tcccagatga cattaccctg ttatccctag atacattacc

361 ctgttatccc agatgacata ccctgttatc cctagatgac attaccctgt tatcccagat

421 gacattaccc tgttatccct agatacatta ccctgttatc ccagatgaca taccctgtta

481 tccctagatg acattaccct gttatcccag ataaactcaa tgatgatgat gatgatggtc

541 gagactcagc ggccgcggtg ccagggcgtg cccttgggct ccccgggcgc gactagttat

601 taatagtaat caattacggg gtcattagtt catagcccat atatggagtt ccgcgttaca

661 taacttacgg taaatggccc gcctggctga ccgcccaacg acccccgccc attgacgtca

721 ataatgacgt atgttcccat agtaacgcca atagggactt tccattgacg tcaatgggtg

781 gagtatttac ggtaaactgc ccacttggca gtacatcaag tgtatcatat gccaagtacg

841 ccccctattg acgtcaatga cggtaaatgg cccgcctggc attatgccca gtacatgacc

901 ttatgggact ttcctacttg gcagtacatc tacgtattag tcatcgctat taccatggtg

961 atgcggtttt ggcagtacat caatgggcgt ggatagcggt ttgactcacg gggatttcca

1021 agtctccacc ccattgacgt caatgggagt ttgttttggc accaaaatca acgggacttc

1081 caaaatgtcg taacaactcc gccccattga cgcaaatggg cggtaggcgt gtacggtggg

1141 aggtctatat aagcagagct cgtttagtga accgtcagat cgcctggaga cgccatccac

1201 gctgttttga cctccataga agacaccggg accgatccag cctccgagat ctgatatctc

1261 tagaccacca tggaagacgc caaaaacata aagaaaggcc cggcgccatt ctatccgctg

1321 gaagatggaa ccgctggaga gcaactgcat aaggctatga agagatacgc cctggttcct

1381 ggaacaattg cttttacaga tgcacatatc gaggtggaca tcacttacgc tgagtacttc

1441 gaaatgtccg ttcggttggc agaagctatg aaacgatatg ggctgaatac aaatcacaga

1501 atcgtcgtat gcagtgaaaa ctctcttcaa ttctttatgc cggtgttggg cgcgttattt

1561 atcggagttg cagttgcgcc cgcgaacgac atttataatg aacgtgaatt gctcaacagt

1621 atgggcattt cgcagcctac cgtggtgttc gtttccaaaa aggggttgca aaaaattttg

1681 aacgtgcaaa aaaagctccc aatcatccaa aaaattatta tcatggattc taaaacggat

1741 taccagggat ttcagtcgat gtacacgttc gtcacatctc atctacctcc cggttttaat

1801 gaatacgatt ttgtgccaga gtccttcgat agggacaaga caattgcact gatcatgaac

1861 tcctctggat ctactggtct gcctaaaggt gtcgctctgc ctcatagaac tgcctgcgtg

1921 agattctcgc atgccagaga tcctattttt ggcaatcaaa tcattccgga tactgcgatt

1981 ttaagtgttg ttccattcca tcacggtttt ggaatgttta ctacactcgg atatttgata

2041 tgtggatttc gagtcgtctt aatgtataga tttgaagaag agctgtttct gaggagcctt

2101 caggattaca agattcaaag tgcgctgctg gtgccaaccc tattctcctt cttcgccaaa

2161 agcactctga ttgacaaata cgatttatct aatttacacg aaattgcttc tggtggcgct

2221 cccctctcta aggaagtcgg ggaagcggtt gccaagaggt tccatctgcc aggtatcagg

2281 caaggatatg ggctcactga gactacatca gctattctga ttacacccga gggggatgat

2341 aaaccgggcg cggtcggtaa agttgttcca ttttttgaag cgaaggttgt ggatctggat

2401 accgggaaaa cgctgggcgt taatcaaaga ggcgaactgt gtgtgagagg tcctatgatt

2461 atgtccggtt atgtaaacaa tccggaagcg accaacgcct tgattgacaa ggatggatgg

2521 ctacattctg gagacatagc ttactgggac gaagacgaac acttcttcat cgttgaccgc

2581 ctgaagtctc tgattaagta caaaggctat caggtggctc ccgctgaatt ggaatccatc

2641 ttgctccaac accccaacat cttcgacgca ggtgtcgcag gtcttcccga cgatgacgcc

2701 ggtgaacttc ccgccgccgt tgttgttttg gagcacggaa agacgatgac ggaaaaagag

2761 atcgtggatt acgtcgccag tcaagtaaca accgcgaaaa agttgcgcgg aggagttgtg

2821 tttgtggacg aagtaccgaa aggtcttacc ggaaaactcg acgcaagaaa aatcagagag

2881 atcctcataa aggccaagaa gggcggaaag atcgccgtgt aattctagag tcggggcggc

2941 cggccgcttc gagcagacat gataagatac attgatgagt ttggacaaac cacaactaga

3001 atgcagtgaa aaaaatgctt tatttgtgaa atttgtgatg ctattgcttt atttgtaacc

3061 attataagct gcaataaaca agttaacaac aacaattgca ttcattttat gtttcaggtt

3121 cagggggagg tgtgggaggt tttttaaagc aagtaaaacc tctacaaatg tggtaaaatc

3181 gataaggatc tgaacgatgg agcggagaat gggcggaact gggcggagtt aggggcggga

3241 tgggcggagt taggggcggg actatggttg ctgactaatt gagatgcatg ctttgcatac

3301 ttctgcctgc tggggagcct ggggactttc cacacctggt tgctgactaa ttgagatgca

3361 tgctttgcat acttctgcct gctggggagc ctggggactt tccacaccct aactgacaca

3421 cattccacag cggatccgtc gaccgatgcc cttgagagcc ttcaacccag tcagctcctt

3481 ccggtgggcg cggggcatga ctatcgtcgc cgcacttatg actgtcttct ttatcatgca

3541 actcgtagga caggtgccgg cagcgctgct cgagagatct acgggtggca tccctgtgac

3601 ccctccccag tgcctctcct ggccctggaa gttgccactc cagtgcccac cagccttgtc

3661 ctaataaaat taagttgcat cattttgtct gactaggtgt ccttctataa tattatgggg

3721 tggagggggg tggtatggag caaggggcaa gttgggaaga caacctgtag ggcctgcggg

3781 gtctattggg aaccaagctg gagtgcagtg gcacaatctt ggctcactgc aatctccgcc

3841 tcctgggttc aagcgattct cctgcctcag cctcccgagt tgttgggatt ccaggcatgc

3901 atgaccaggc tcagctaatt tttgtttttt tggtagagac ggggtttcac catattggcc

3961 aggctggtct ccaactccta atctcaggtg atctacccac cttggcctcc caaattgctg

4021 ggattacagg cgtgaaccac tgctcccttc cctgtccttt ctagagtcga cccatggggg

4081 cccgccccaa ctggggtaac ctttgagttc tctcagttgg gggtaatcag catcatgatg

4141 tggtaccaca tcatgatgct gattataaga atgcggccgc cacactctag tggatctcga

4201 gttaataatt cagaagaact cgtcaagaag gcgatagaag gcgatgcgct gcgaatcggg

4261 agcggcgata ccgtaaagca cgaggaagcg gtcagcccat tcgccgccaa gctcttcagc

4321 aatatcacgg gtagccaacg ctatgtcctg atagcggtcc gccacaccca gccggccaca

4381 gtcgatgaat ccagaaaagc ggccattttc caccatgata ttcggcaagc aggcatcgcc

4441 atgggtcacg acgagatcct cgccgtcggg catgctcgcc ttgagcctgg cgaacagttc

4501 ggctggcgcg agcccctgat gctcttcgtc cagatcatcc tgatcgacaa gaccggcttc

4561 catccgagta cgtgctcgct cgatgcgatg tttcgcttgg tggtcgaatg ggcaggtagc

4621 cggatcaagc gtatgcagcc gccgcattgc atcagccatg atggatactt tctcggcagg

4681 agcaaggtga gatgacagga gatcctgccc cggcacttcg cccaatagca gccagtccct

4741 tcccgcttca gtgacaacgt cgagcacagc tgcgcaagga acgcccgtcg tggccagcca

4801 cgatagccgc gctgcctcgt cttgcagttc attcanggca ccggacaggt cggtcttgac

4861 aaaaagaacc gggcgcccct gcgctgacag ccgggancac ggcggcatca gnncagccga

4921 ttgtnntgtt gtgcccagtt catannccga atagcctctc cacccaagcc ggccgganna

4981 anctgcgtgc aatccatctt ngntnnntca tgcgaaangn nnnnnnnnna aaaggccgcg

5041 ttgctggcgt ttttccatag gctccgcccc cctgacgagc atcacaaaaa tcgacgctca

5101 agtcagaggt ggcgaaaccc gacaggacta taaagatacc aggcgtttcc ccctggaagc

5161 tccctcgtgc gctctcctgt tccgaccctg ccgcttaccg gatacctgtc cgcctttctc

5221 ccttcgggaa gcgtggcgct ttctcatagc tcacgctgta ggtatctcag ttcggtgtag

5281 gtcgttcgct ccaagctggg ctgtgtgcac gaaccccccg ttcagcccga ccgctgcgcc

5341 ttatccggta actatcgtct tgagtccaac ccggtaagac acgacttatc gccactggca

5401 gcagccactg gtaacaggat tagcagagcg aggtatgtag gcggtgctac agagttcttg

5461 aagtggtggc ctaactacgg ctacactaga aggacagtat ttggtatctg cgctctgctg

5521 aagccagtta ccttcggaaa aagagttggt agctcttgat ccggcaaaca aaccaccgct

5581 ggtagcggtg gtttttttgt ttgcaagcag cagattacgc gcagaaaaaa aggatctcaa

5641 gaagatcctt tgatcttttc tacggggtct gacgctcagt ggaacgaaaa ctcacgttaa

5701 gggattttgg tcatgagatt atcaaaaagg atcttcacct agatcctttt aaattaaaaa

5761 tgaagtttta aatcaatcta aagtatatat gagtaaactt ggtctgacag ttaccaatgc

5821 ttaatcagtg aggcacctat ctcagcgatc tgtctatttc gttcatccat agttgcctga

5881 ctccccgtcg tgtagataac tacgatacgg gagggcttac catctggccc cagtgctgca

5941 atgataccgc gagacccacg ctcaccggct ccagatttat cagcaataaa ccagccagcc

6001 ggaagggccg agcgcagaag tggtcctgca actttatccg cctccatcca gtctattaat

6061 tgttgccggg aagctagagt aagtagttcg ccagttaata gtttgcgcaa cgttgttgnn

6121 nnaaaaagga tcttcaccta gatccttttc acgtagaaag ccagtccgca gaaacggtgc

6181 tgaccccgga tgaatgtcag ctactgggct atctggacaa gggaaaacgc aagcgcaaag

6241 agaaagcagg tagcttgcag tgggcttaca tggcgatagc tagactgggc ggttttatgg

6301 acagcaagcg aaccggaatt gccagctggg gcgccctctg gtaaggttgg gaagccctgc

6361 aaagtaaact ggatggcttt ctcgccgcca aggatctgat ggcgcagggg atcaagctct

6421 gatcaagaga caggatgagg atcgtttcgc atgattgaac aagatggatt gcacgcaggt

6481 tctccggccg cttgggtgga gaggctattc ggctatgact gggcacaaca gacaatcggc

6541 tgctctgatg ccgccgtgtt ccggctgtca gcgcaggggc gcccggttct ttttgtcaag

6601 accgacctgt ccggtgccct gaatgaactg caagacgagg cagcgcggct atcgtggctg

6661 gccacgacgg gcgttccttg cgcagctgtg ctcgacgttg tcactgaagc gggaagggac

6721 tggctgctat tgggcgaagt gccggggcag gatctcctgt catctcacct tgctcctgcc

6781 gagaaagtat ccatcatggc tgatgcaatg cggcggctgc atacgcttga tccggctacc

6841 tgcccattcg accaccaagc gaaacatcgc atcgagcgag cacgtactcg gatggaagcc

6901 ggtcttgtcg atcaggatga tctggacgaa gagcatcagg ggctcgcgcc agccgaactg

6961 ttcgccaggc tcaaggcgag catgcccgac ggcgaggatc tcgtcgtgac ccatggcgat

7021 gcctgcttgc cgaatatcat ggtggaaaat ggccgctttt ctggattcat cgactgtggc

7081 cggctgggtg tggcggaccg ctatcaggac atagcgttgg ctacccgtga tattgctgaa

7141 gagcttggcg gcgaatgggc tgaccgcttc ctcgtgcttt acggtatcgc cgctcccgat

7201 tcgcagcgca tcgccttcta tcgccttctt gacgagttct tctgaatttt gttaaaattt

7261 ttgttaaatc agctcatttt ttaaccaata ggccgaaatc ggcaacatcc cttataaatc

7321 aaaagaatag accgcgatag ggttgagtgt tgttccagtt tggaacaaga gtccactatt

7381 aaagaacgtg gactccaacg tcaaagggcg aaaaaccgtc tatcagggcg atggcccact

7441 acgtgaacca tcacccaaat caagtttttt gcggtcgagg tgccgtaaag ctctaaatcg

7501 gaaccctaaa gggagccccc gatttagagc ttgacgggga aagccggcga acgtggcgag

7561 aaaggaaggg aagaaagcga aaggagcggg cgctagggcg ctggcaagtg tagcggtcac

7621 gctgcgcgta accaccnnnn nnnnnnnnnn nnnnnnnnnn nnnnnnnnnn nnnnnnnnnn

7681 nnnnnnnnnn nnnnnnnnnn nnnnnnnnnn
